# Quality indicators and performance measures for prison healthcare: a scoping review

**DOI:** 10.1186/s40352-022-00175-9

**Published:** 2022-03-07

**Authors:** Sue Bellass, Krysia Canvin, Kate McLintock, Nat Wright, Tracey Farragher, Robbie Foy, Laura Sheard

**Affiliations:** 1 Leeds Institute for Health Sciences, University of Leeds, Leeds, UK; 2 Spectrum Community Health CIC, Wakefield, UK; 3 Division of Population Health, Health Services Research & Primary Care, University of Manchester, Manchester, UK; 4 York Trials Unit, University of York, York, UK

**Keywords:** Quality indicators, Performance measurement, Prison healthcare, Correctional healthcare, Quality of prison healthcare

## Abstract

**Background:**

Internationally, people in prison should receive a standard of healthcare provision equivalent to people living in the community. Yet efforts to assess the quality of healthcare through the use of quality indicators or performance measures have been much more widely reported in the community than in the prison setting. This review aims to provide an overview of research undertaken to develop quality indicators suitable for prison healthcare.

**Methods:**

An international scoping review of articles published in English was conducted between 2004 and 2021. Searches of six electronic databases (MEDLINE, CINAHL, Scopus, Embase, PsycInfo and Criminal Justice Abstracts) were supplemented with journal searches, author searches and forwards and backwards citation tracking.

**Results:**

Twelve articles were included in the review, all of which were from the United States. Quality indicator selection processes varied in rigour, and there was no evidence of patient involvement in consultation activities. Selected indicators predominantly measured healthcare processes rather than health outcomes or healthcare structure. Difficulties identified in developing performance measures for the prison setting included resource constraints, data system functionality, and the comparability of the prison population to the non-incarcerated population.

**Conclusions:**

Selecting performance measures for healthcare that are evidence-based, relevant to the population and feasible requires rigorous and transparent processes. Balanced sets of indicators for prison healthcare need to reflect prison population trends, be operable within data systems and be aligned with equivalence principles. More effort needs to be made to meaningfully engage people with lived experience in stakeholder consultations on prison healthcare quality. Monitoring healthcare structure, processes and outcomes in prison settings will provide evidence to improve care quality with the aim of reducing health inequalities experienced by people living in prison.

**Supplementary Information:**

The online version contains supplementary material available at 10.1186/s40352-022-00175-9.

## Background

In 2018 the number of people in penal institutions worldwide was at least 10.74 million, an average of around 140 people per 100,000 of the world’s population (Walmsley, 2018). Although epidemiological data is limited in some countries (Kinner & Young, 2018), evidence suggests that people who experience incarceration are more likely to be disproportionately impacted by structural health inequalities than those who have not lived in prison (Brinkley-Rubinstein, 2013; De Viggiani, 2007). Mental health problems, substance misuse, cognitive disability, communicable and non-communicable diseases (Fazel, Hayes, Bartellas, Clerici, & Trestman, 2016; Stürup-Toft, O’Moore, & Plugge, 2018; Thomas, Wang, Curry, & Chen, 2016; Tyler, Miles, Karadag, & Rogers, 2019; World Health Organisation, 2019), alongside lower levels of health service engagement (Begun, Early, & Hodge, 2016; Hopkin, Evans-Lacko, Forrester, Shaw, & Thornicroft, 2018), are more prevalent amongst people who have experienced incarceration.

Since reducing health inequalities is a fundamental principle of global public health policies (Stürup-Toft et al., 2018), there is a clear imperative to address the complex health needs of prison populations. Statutory responsibilities towards the human rights of prisoners – including their health - are outlined in the United Nations’ Standard Minimum Rules for Treatment of Prisoners (known as the Nelson Mandela Rules), which state that people living in prison ‘should enjoy the same standards of health care that are available in the community’ (Rule 24.1, a stance known as the equivalence principle) and that prison healthcare services are responsible for ‘evaluating, promoting, protecting and improving’ the health of incarcerated people (Rule 25.1) (United Nations General Assembly, 2015). Thus prison, which represents an opportunity to improve the health of underserved populations (Ginn, 2013; McLeod et al., 2020), is charged with dual and related objectives of providing equivalent care (healthcare process) and improving health (health outcomes). Yet whether the provision of an equivalent standard of care - given the health inequities between the prison population and the population as a whole - will reduce inequalities satisfactorily is a matter of some debate. Several authors have argued that the primary goal of prison healthcare should be a reduction in health inequities through greater, rather than equal, intensity of service provision (Birmingham, Wilson, & Adshead, 2006; Charles & Draper, 2012; Exworthy, Wilson, & Forrester, 2011; Ismail & de Viggiani, 2018; Jotterand & Wangmo, 2014; Lines, 2006; Niveau, 2007). What is not disputed is that - whichever goal is given primacy - prison healthcare globally needs to generate reliable evidence on healthcare provision and to be more accountable (McLeod et al., 2020). This could be facilitated in part by the implementation of transparent monitoring systems to measure evidence-based performance of prison healthcare and identify areas for improvement (Asch et al., 2011; Greifinger, 2012; Halachmi, 2002; Mainz, 2003). Such performance measurement would enable regular internal analyses of the quality of healthcare within a single prison, and permit external comparisons with healthcare provided in other prison establishments and in the community.

Selecting appropriate measures of performance, however, is not unproblematic (Kötter, Blozik, & Scherer, 2012; Loeb, 2004). There may be more than one set of evidence-based standards from which to develop quality indicators (Castro, 2014; Greenhalgh, Howick, & Maskrey, 2014; Willis et al., 2017), or, as was the case until recently for women in prison, a dearth of rigorously developed standards (McCann, Peden, Phipps, Plugge, & O’Moore, 2019). Translating an evidence-based standard into a quantifiable measure involves multiple decisions and this process is often poorly reported (Kötter et al., 2012). Additionally, resource constraints limit the collection and analysis of data to a relatively small number of indicators, which inevitably privileges some health conditions and, by extension, some populations over others; decisions therefore have to be made regarding the potential for positive impact for patients (Rushforth et al., 2015) with some stakeholders inevitably having more input into the selection process than others. Further, due to the unique nature of delivering healthcare in prison, some quality indicators may not be able to be simply taken from community primary care and “parachuted” into the prison setting due to significant differences in disease prevalence, premature physiological ageing (Omolade, 2014; Williams, Stern, Mellow, Safer, & Greifinger, 2012), the short periods of time many people are incarcerated for and the limited functionality for linkage between community and prison clinical systems (Stone, Kaiser, & Mantese, 2006). Therefore, it is essential to explore the challenges particular to measuring performance in this context. The aim of this international scoping review is to identify and synthesise previous research conducted on the selection and development of quality indicators in the prison setting.

## Methods

A scoping review is a method of mapping the conceptual terrain of a particular topic (Arksey & O’Malley, 2005; Peters et al., 2020; Tricco et al., 2016). In comparison to systematic reviews, which aim to synthesise evidence on specific questions often relating to interventions, scoping reviews explore the breadth and depth of available literature, define key concepts, outline methodological approaches and identify knowledge gaps. As such, scoping reviews tend to have broad research questions, and take an inclusive stance towards evidence sources. Although scoping review methodology has historically been poorly defined in comparison to systematic reviews, recent efforts to standardise scoping reviews has resulted in the establishment of the PRISMA-ScR, a reporting checklist (Tricco et al., 2018). The conduct of this study has been guided by the items on the PRISMA-ScR. The research question for this study is:

What is known from the research literature about the development and selection of quality indicators for primary healthcare in the prison setting?

### Study selection

The focus for this international review was the development or selection of quality indicators for healthcare within the prison context. Papers that focussed on the transition of people between prison and the community, or healthcare delivery in criminal justice settings in the community were excluded.

We searched six databases that we anticipated would index relevant sources: CINAHL and Criminal Justice Abstracts (via the Ebsco platform), MEDLINE, PsycInfo and Embase (via the Ovid platform) and Scopus, from January 2004 to April 2021. 2004 was chosen as the start date as it marked the beginning of the prison healthcare governance transition from the Home Office to the National Health Service in the UK, and was also a time when authors were reflecting on growing accountability and strategic management models in prison systems in other countries (Coyle, 2004; K. N. Wright, 2005). The electronic database search strategy was informed by a published search strategy on primary care, quality indicators and severe mental illness (Kronenberg et al., 2017) and was constructed around three key concepts: quality indicators/ performance measurement, primary care and prison healthcare. An academic librarian developed the search syntax (see Appendix for a sample search strategy). Research papers, commentaries, editorials and grey literature were included. Since the purpose of this review was to provide a descriptive overview of the body of literature on quality indicators in the prison setting, rather than to assess the robustness of clinical evidence underpinning quality indicators, sources were not subjected to critical appraisal.

### Supplementary searches

Three supplementary search strategies were employed: journal searches, author searches, and forwards and backwards citation tracking. The five journals handsearched from January 2004 to April 2021 were: *International Journal of Prisoner Health*, *Journal of Correctional Health Care*, *British Journal of General Practice*, *BMC Health & Justice* (from Volume 1, 2013) and *The Prison Journal*. Author searches, and forwards and backwards citation tracking were conducted following identification of key papers.

### Search results

**Fig. 1:**
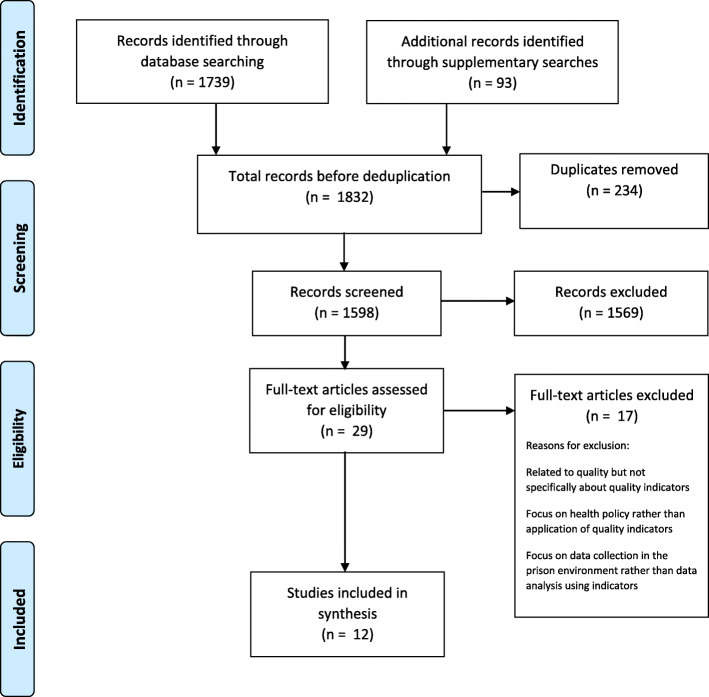
PRISMA flow diagram

The electronic search returned 1739 hits. A further 93 sources were identified through the supplementary searches. Following automated and manual deduplication of the combined total of 1832 sources, 1598 unique sources were available for screening (see Fig. 1). Title, abstract and full-text screening was conducted independently by two researchers (SB and KC), using inclusion and exclusion criteria listed in Table 1, with each reviewing the other’s exclusions, and any disputes were resolved in discussion with a third member of the team (LS).

**Table 1:** Inclusion and Exclusion Criteria

Inclusion criteria	Exclusion criteria
Any type of literature that relates to the selection, development or review of quality indicators in the prison setting (either adult or juvenile)	Literature relating to criminal justice settings in the community
Any research method employed (empirical papers) Relating to any health condition	Literature relating to the transition from prison to community healthcare settings
Published between January 2004 and April 2021; English Language only International literature	Literature published in a language other than English

Twelve sources from the United States were included in the review (Table 2); no sources from any other country were identified. The date range of sources was 2004–2016. Three of the publications, Asch et al. (2011), Teleki et al. (2011) and Damberg et al. (2011), were part of the same research project: all were published in a special issue of the *Journal of Correctional Health Care*. The study was organised into three workpackages: an expert consultation process reported in Asch et al., with the resulting list of indicators published by Teleki et al., interviews, site visits and document reviews within California Department of Corrections and Rehabilitation (Teleki et al., 2011), and a review of performance measurement activities in six correctional systems (Damberg, Shaw, Teleki, Hiatt, & Asch, 2011). None of the remaining sources were linked to each other.

**Table 2:** Sources included in the review

Author(s)/ title/ journal	Year	Country	Type of source/ study design	Develops, reviews, tests quality indicators / performance measures	Presents list of quality indicators or performance measures	Key findings
Raimer & Stobo Health care delivery in the Texas prison system: the role of academic medicine *Journal of the American Medical Association*	2004	USA	Report of a healthcare delivery system in Texas prison system	Develops and implements	**√**	The implementation of a managed care programme improved the care for 6 chronic conditions and improved health outcomes including blood glucose for people with diabetes and low density lipoprotein levels for people with hyperlipidaemia
Wright Designing a national performance measurement system *The Prison Journal*	2005	USA	Reports on the development of correctional measures for all aspects of the prison system including health	Develops	**√**	Consensus process identified 8 domains as priorities for the prison population, including ‘health’ and ‘substance abuse and mental health’
Stone et al. Health care quality in prisons: a comprehensive matrix for evaluation *Journal of Correctional Health Care*	2006	USA	Development of a quality indicator matrix for the Missouri Department of Corrections	Develops	**√**	Identified 32 quality indicators for correctional health distributed across 11 health domains, including women’s health, infectious disease and long term conditions
Hoge et al. Mental health performance measurement in corrections *International Journal of Offender Therapy & Comparative Criminology*	2009	USA	Consensus panel development of mental health service performance measures	Develops	**√**	Four key areas of mental health care in prisons were identified as priorities - medication adherence, suicide prevention, mental health treatment planning, and sleep medication usage. Standards-based performance measures were recommended for each area.
Asch et al. Selecting Performance Indicators for Prison Health Care *Journal of Correctional Health Care*	2011	USA	Modified Delphi method to select quality indicators	Develops	**√**	79 quality indicators were retained relating to a range of clinical areas including medication monitoring, infectious disease, psychiatric disorders and substance use and metabolic diseases
Damberg et al. A Review of Quality Measures Used by State and Federal Prisons *Journal of Correctional Health Care*	2011	USA	Review of quality indicators used in six US correctional health systems	Reviews		Observed considerable variation across the systems’ performance measurement portfolios and data system functionality, although all included explicit quality indicators, prevalence measures and standards covering a broad spectrum of health conditions.
Teleki et al. The Current State of Quality of Care Measurement in the California Department of Corrections and Rehabilitation *Journal of Correctional Health Care*	2011	USA	Review of the approaches to performance measurement in California Department of Corrections	Reviews	**√**	Identified gaps in quality measurement, including the measurement of access, clinical quality and the measurement of people’s experience of care provision
Bisset & Harrison Health outcomes in corrections: health information technology and the correctional health outcome and resource data set *Community Oriented Correctional Health Services*	2012	USA	Pilot study of a national data set to monitor quality in US prisons	Develops and tests	**√**	A national US data set for corrections will establish a benchmarking system where quality indicators can be standardised allowing facilities to assess their performance in comparison to others.
Greifinger Independent review of clinical health services for prisoners *International Journal of Prisoner Health*	2012	USA	Proposes a systematic method for assessing the quality of health care in the prison setting	Develops	**√**	The quality of clinical care in the prison setting can be assessed with reference to performance measures covering a range of health domains including suicide prevention, medication management and chronic health conditions.
Castro Diabetes screening in inmates: a quality improvement pilot project *Dissertation*	2014	USA	Expert consultation to select a guideline to reduce the risk of type 2 diabetes	Develops and tests		A clinical panel selected a diabetes screening guideline to facilitate performance measurement of diabetes screening
Watts Development of a Performance-Based RFP for Correctional Health Care Services in Vermont *Community Oriented Correctional Health Services*	2015	USA	Development of performance metrics for Vermont Department of Corrections	Develops and tests	**√**	The development of evidence-based performance measures that align with policy and reform allows for benchmarking both within prison systems and between prisons and the community
Laffan Evaluation of your medical department *American Jails*	2016	USA	Commentary recommending the implementation of quality assessment in prison healthcare departments	Advises on implementation	**√**	An effective performance measurement system depends upon the efficiency of the working relationship between the medical team and the correctional administrators.

### Data charting

A data charting table was constructed using generic study features informed by the Joanna Briggs Institute Manual for Evidence Synthesis (Peters et al., 2020). Bespoke elements were integrated iteratively following detailed reading of the texts selected after full-text review. The table was constructed by one researcher (SB) and reviewed by two others (KC and LS).

Data items relating to the features of the study were extracted, such as the country of origin, the year, the study type, study aims and key findings. In addition, contextual elements relating to the development of quality indicators were charted, including drivers for the development of performance measurement, the challenges and constraints of the prison environment, issues relating to the transfer of performance measures from a community setting, and stakeholder engagement in decision-making processes.

### Findings

Five studies developed quality indicators or performance measures (Asch et al., 2011; Greifinger, 2012; Hoge, B., Lundquist, & Mellow, 2009; Stone et al., 2006; K. N. Wright, 2005), two sources reviewed indicators or approaches to performance measurement (Damberg et al., 2011; Teleki et al., 2011), one described implementation (Raimer & Stobo, 2004), and one commentary paper advised on implementation (Laffan, 2016). Two sources described approaches to developing and testing indicators across US prisons (Bisset & Harrison, 2012; Watts, 2015), and one developed then tested performance measures of diabetes screening in one prison (Castro, 2014).

### Quality indicators and performance measures for the prison setting

Several papers in the review described methods of selecting performance measures or quality indicators (Asch et al., 2011; Greifinger, 2012; Hoge et al., 2009; Stone et al., 2006; Watts, 2015; K. N. Wright, 2005), with the quality indicators resulting from Asch et al.’s (Asch et al., 2011) consultation process being reported in sister paper Teleki et al. (2011). Issues raised by the authors of this group of papers include interrelated notions of comparability and transferability, that is, the extent to which the prison population has comparable health needs and health behaviours to people living in the community, whether the prison health care setting bears similarity to those in the community, and hence whether indicators from community health care settings have ‘external validity’ (Stone et al., 2006, p.94) and can reasonably be transferred with the same benchmarks to the prison setting. An additional area of interest relates to the extent to which each criminal justice setting should be able to customise recommended indicators which align with their mission statements and priorities, despite the impact this would have on standardisation and benchmarking, and which stakeholder voices are privileged in selection processes, and which unheard. Finally, pragmatism was observed to be an important aspect of quality measurement; staff and IT resources constrain the number of indicators it is practicable to collect and analyse data for.

#### Processes of selecting performance measures and quality indicators

Greifinger’s (2012) performance measures are orientated towards improving the safety of people living in prison. Drawing on national and international prison healthcare standards, community patient safety standards relevant to the prison setting, and his own experience of reviewing correctional healthcare, he compiled a guide of measures covering 30 domains of prison healthcare, including (but not limited to) access to care, chronic disease management, mental health assessment and treatment, medical record keeping, sexually transmitted infections, and mortality reviews.

In contrast to this individual approach to compiling performance measures, other authors described consensus approaches to indicator selection. Asch et al. (2011), for instance, utilised a modified Delphi method, drawing on the expertise of a panel comprising nine senior people with clinical experience in correctional healthcare as well as relevant experience in other areas such as prison directorships, court-appointed monitorships and membership of clinical guideline committees. Following preparatory investigations (Damberg et al., 2011; Teleki et al., 2011), 16 healthcare topics were chosen for further investigation, and 1069 relevant indicators were identified and classified using Donabedian’s structure-process-outcome taxonomy (Donabedian, 1988). Content reviewers evaluated groups of indicators using criteria including importance to prison health care, focus on primary care, scientific evidence base, implementability and interpretability. As a result of this process, 111 indicators were presented to the panel for validity and feasibility assessment, with a 0–9 rating requested from panel members both before and during the meeting. Ultimately, 79 measures were retained, 62 of which were process indicators, 10 outcome indicators and 7 access indicators. The panel remarked that while these quantitative measures were valuable means of assessing quality, they needed to be augmented by implicit quality measures such as mortality reviews and patient experience surveys for a more comprehensive view of prison healthcare quality. Processes to select guidelines, perform content reviews, and engage an expert panel for the selection process were clearly articulated; the expertise of the reviewers was described and the rationales for selection and elimination of indicators were coherent. However, testing and implementing the measures was beyond the scope of the study, and it is possible that a set of 79 indicators, in an environment where requirements for data collection for purposes other than quality assessment can be onerous (Teleki et al., 2011), may be too burdensome.

While others have used consultation methods to identify quality indicators and performance measures, none match Asch et al.’s (2011) rigorous multi-staged approach. Stone et al. (2006), for instance, in their development of a quality indicator matrix for the Missouri Department of Corrections, appeared to rely only on the research team to identify the domains of healthcare delivery for which to identify standards and quality indicators, although administrators and medical staff were involved in selecting the final 32 indicators from an original list of 150. Where Stone et al.’s (2006) work differed from Asch et al.’s (2011) was in their attempts to define performance benchmarks based on community benchmark data for similar indicators. This involved some modification of the indicators, for example, age range adjustments, to more closely align the prison population – often perceived as prematurely aged (Omolade, 2014; Williams et al., 2012) - with the population as a whole.

Another study that sought to adapt community indicators for the prison setting was Hoge et al.’s (Hoge et al., 2009) selection of performance measures for mental health care in prisons. Twenty nine participants including for-profit and independent mental health practitioners and researchers participated in a 6-hour roundtable discussion to reach consensus on meaningful indicators drawn from national standards. According to the authors, consensus was reached on nearly every subject, but how ‘consensus’ was defined and assessed is not clearly articulated.

Watts (2015) reports on the development of a quality indicator set based on the Healthcare Effectiveness Data and Information Set (HEDIS®) metrics, the work conducted by the RAND organisation in 2011 (Asch et al., 2011; Damberg et al., 2011; Teleki et al., 2011) and the Vermont Department of Corrections internal measurement system. However, very little information is given on the processes through which some of the measures were adapted for the prison setting. Similarly, Laffan (2016), Bisset and Harrison (2012) and Raimer and Stobo (2004) provide short lists of measures but only minimal detail on the origin or development of the indicators.

Wright (2005) recounts the Association of State Correctional Administrators’ (ASCA) preliminary efforts to identify eight domains across the spectrum of activities in correctional systems that could be subject to a national performance measurement system, enabling a greater degree of transparency and accountability. Using seven comprehensive prison performance models, an ASCA subcommittee selected the eight most pertinent areas of correctional performance to assess, two of which were health-related: ‘substance abuse and mental health’ and ‘health’. The subcommittee then selected three of the eight for their preliminary performance measurement system, including ‘substance abuse and mental health’ but excluding ‘health’. Following some debate, the subcommittee decided upon performance indicators for each domain; for substance abuse and mental health, they chose average daily rates of people receiving treatment for both conditions to be the indicators of performance.

In all of the papers that developed or selected indicators in this review, none explicitly included the patient perspective, drawing instead on researcher, healthcare provider or manager input. However, it was noted by one group of authors, Asch et al. (2011), that people on the receiving end of care may have different priorities for performance measures, perhaps placing more value on outcome indicators which measure changes in health status or highlight risks of mortality, than those relating to healthcare processes.

Processes used to identify performance measures or quality indicators for the prison setting are summarised in Table 3.

**Table 3:** Processes used to identify quality indicators

Author(s) and year	Domains of health or healthcare included	Consensus/ expert consultation process	Sources of indicators	Inclusion criteria	Exclusion criteria	Number of indicators	Benchmarking / performance targets
**Wright (**2005**)**	Substance abuse and mental health	Yes	Association of State Correctional Administrators subcommittee	• Detailed • Valid • Allows for comparison/ is applicable across different organisations • Manageable number	Not explicitly stated	2	• Benchmarking requires inter-agency performance partnerships • Rates per population unit rather than numbers provided to enable cross-agency comparison • Clearly defined numerators and denominators with counting guidance should be provided
**Stone et al. (**2006**)**	*Healthcare delivery areas:* • Acute • Subacute • Ambulatory • Behavioural health • Nursing/assisted living • Rehabilitation • Hospice care *Health categories:* • Women’s health • Heart disease • Infectious disease • Pulmonary disease • Wellness and prevention • Asthma • Diabetes • Medication administration • Screening • Behavioural health	Yes	4 US national public and private agency guidelines including prison-specific and community guidelines	• Relevant to health needs of the prison population • Addressed the range of care services in the correctional system • Addresses health issues that are amenable to change • Evidence-based • Relevant to specific organisational goals/ policy directives • Has available and reliable data, analysable at different levels e.g. organisation, gender, age etc. • Contributes to balanced coherent set of measures	• Indicators requiring intensive data collection • Indicators with sporadic / unreliable data • Indicators reliant on subjective judgements • Community indicators that could not be modified for a prison population	32	• Benchmarks selected on available data for most comparable community population
**Hoge et al. (**2009**)**	*Mental health:* • Medication adherence • Monitoring for side effects and toxicity • Suicide prevention • Treatment planning • Sleep medication	Yes	Unspecified number of prison- and condition-specific guidelines	• Meaningful • Must be quantitative to allow for analysis of longitudinal trends • Based on nationally accepted standards	Not explicitly stated	26	Not explicitly stated
**Asch et al. (**2011**)**	• Access • Cardiac conditions • Geriatrics • Infectious diseases • Medication monitoring • Metabolic diseases • Obstetrics/gynecology • Screening/prevention • Psychiatric disorders/ substance abuse • Pulmonary conditions • Urgent conditions	Yes	29 national and international prison- and condition-specific guidelines	*Content reviewer criteria*: • Importance • Scientific soundness • Implementability • Interpretability *Panellist criteria (0–9 scale, median score used):* a) **Validity** • Soundness of evidence • Identifiable health benefits • Compliance would indicate high quality provision • Compliance is under control of prison healthcare b) **Feasibility** • Data can be readily extracted • Reliable, unbiased, consistent data • Failure to document data is an indicator of poor quality	• Inpatient/ specialist care because not under control of prison healthcare • Indicators scoring < 7 on validity and < 4 on feasibility	79	Not explicitly stated
**Greifinger (**2012**)**	• Suicide screening • Health assessments • Urgent care • Obstetrics/ gynaecology • Infectious disease • Medication administration and continuity • Access to care • Chronic disease care • Anticoagulant medication • Side-effect monitoring • Transfer planning • Dental care • Credentialing • Grievance reporting • Inclusivity	No	Unspecified number of international, national prison- and condition-specific guidelines and guidelines produced by independent authors	• Potential to improve patient safety through reducing risk of harm • Focus on areas where most serious harm could be caused through non-adherence to the measure • Quantitative measures allowing comparative analysis	• Outcome measurement such as rates of mortality and preventable infections because difficult to provide meaningful measurement in small populations	30 (with sub-items)	• Expected performance measure 90%, although some measures should be 100% • Comparative analysis of facilities identifies areas for improvement
**Watts (**2015**)**		Yes (though limited detail)	Healthcare Effectiveness Data and Information Set metrics (HEDIS®, developed by the National Committee for Quality Assurance), the indicators listed in Teleki et al. (Teleki et al., 2011), and Vermont DOC’s reporting requirements	• HEDIS® measures that could be adapted for the prison setting • Improvement in people’s health status given more priority than care processes in results-based accountability • Focus on chronic care management	Not explicitly stated	53	• More robust health record data set was required to interface correctional data with community data enabling comparative analysis and continuity of care • Pay-for-performance contract set expected performance at 85% in first year, rising by 5% in the second and third years

#### Identifying the problem and benchmarking

Setting performance targets for quality indicators to enable meaningful benchmarking has been less well developed in this body of literature. Stone et al., in their 2006 development of a matrix of prison healthcare quality indicators, modified community healthcare quality indicators to facilitate comparison between prison and community healthcare. Greifinger (2012) set a 90% target for the majority of his performance indicators, yet the rationale for settling on this figure was not evident; similarly, Watts (2015) suggested an 85% target, rising to 90% by the second year and 95% by the third, again with no rationale given. Other authors, while providing clearly delineated numerators and denominators, did not suggest what an acceptable level of performance would be.

#### Format of quality indicators and performance measures used in the prison setting

Most of the literature included in the review listed quality indicators or performance measures, although the content varied widely from a few illustrative examples (Asch et al., 2011; Bisset & Harrison, 2012; Laffan, 2016; Raimer & Stobo, 2004; K. N. Wright, 2005) to extensive lists (Greifinger, 2012; Hoge et al., 2009; Stone et al., 2006; Teleki et al., 2011; Watts, 2015). Further variation was found in the format of measures, with some authors providing ‘explicit’ quality indicators (Asch et al., 2011; Raimer & Stobo, 2004; Stone et al., 2006; Teleki et al., 2011; Watts, 2015) - defined by Damberg et al. (2011) as objective, evidence-based measures that provide a standardised means of measuring quality across prisons - while others provided more broadly stated performance measures (Bisset & Harrison, 2012; Greifinger, 2012; Hoge et al., 2009; Laffan, 2016; K. N. Wright, 2005). Explicit indicators, Damberg et al. suggest, are distinguishable by their format; they have a clearly expressed denominator i.e. the number of people eligible for a particular measure, and a specified numerator i.e. the number of people from the denominator who satisfy the measure. Further parameters are often included, such as a reporting period (for example, the last 12 months) or particular diagnostic codes. The measure is then expressed as a percentage, calculated by dividing the numerator by the denominator and multiplying by 100. Explicit quality indicators typically fall into one of three classifications: ‘structure’ indicators, relating to resources, ‘process’ indicators, focussing on care delivery, or ‘outcome’ indicators, which measure the achievement of a particular health outcome (Donabedian, 1988), as shown in Table 4.

**Table 4:** Examples of structure, process and outcome explicit quality indicators

**Structure indicator**	
**Numerator**	Number of nurses in the establishment with recognised diabetes training
**Denominator**	Total number of nurses in the reporting period
**Process indicator**	
**Numerator**	Number of prisoners from the denominator who received at least one serum potassium and either a serum creatinine (Cr) or a blood urea nitrogen (BUN) therapeutic monitoring test in the measurement year
**Denominator**	Total number of prisoners who received at least a 180-day supply of ACEIs, ARBs, or diuretics during the measurement year (Teleki et al., 2011)
**Outcome indicator**	
**Numerator**	Number of prisoners from the denominator having Low Density Lipoprotein < 100 on or between 60 and 365 days after discharge for an acute cardiovascular event
**Denominator**	Total number of prisoners age 18 to 75 years as of 12/31 of the reporting year who were discharged alive in the year before the reporting year for acute myocardial infarction (Teleki et al., 2011)

In the reviewed body of literature, performance measures provided ways to assess prison healthcare quality, but the numerators, denominators and reporting periods were typically implied rather than specified. For example, Greifinger (2012) appended a list of questions that could identify areas for clinical performance improvement through the interrogation of randomly-selected small samples of healthcare records. For example, taking ten records of people with positive tests for syphilis, gonorrhoea and chlamydia, Greifinger suggested that a measure of quality would be those who had received an appropriate prescription to treat their condition within 3 days. Similarly, Hoge et al. (2009) suggested that people in prison who screen positive on a validated suicide risk assessment measure should ‘receive a referral to a mental health staff member for evaluation. All inmates deemed to be an acute risk should be placed on suicide watch immediately and be immediately referred to the mental health team’ (p.643). Thus, the numerators and denominators are implicit in these measures of healthcare quality, but further work would be required to clarify the parameters of the metrics before they could be implemented in practice; clarifying denominators in the prison population, for instance, is particularly challenging given the transience of the population as people move between the community and the prison estate or are transferred between prisons.

To create a concise list, and following Damberg et al. (2011) and Kronenberg et al.’s (2017) lead, quality indicators and performance measures identified in the sources have been merged and summarised under broad headings in Table 5.

**Table 5:** Quality indicators and performance measures identified for use in the prison setting

	Source
**SECTION 1: HEALTHCARE**	
**1. Routine health assessments**	
Percentage of people receiving physical health examination within first week following admission	(Greifinger, 2012; Laffan, 2016; Stone et al., 2006; Watts, 2015)
Percentage of people dental examination within first month following admission	(Stone et al., 2006; Watts, 2015)
Percentage of people receiving a mental health evaluation within 24 h of admission	(Teleki et al., 2011; Watts, 2015)
**2. Access to care**	
Percentage of people with urgent sick calls accessing primary care the same or the following day	(Teleki et al., 2011; Watts, 2015)
Percentage of people referred to urgent specialty care accessing care within 14 days	(Teleki et al., 2011)
Percentage of people with non-urgent sick calls being seen within 48–72 h (Watts) or 14 days (Teleki)	(Teleki et al., 2011; Watts, 2015)
**3. Infectious diseases**	
Receipt of influenza vaccination for people deemed high-risk (age and/or chronic conditions)	(Greifinger, 2012; Stone et al., 2006; Teleki et al., 2011)
Receipt of pneumonia vaccination for people deemed high-risk (age and/ or chronic conditions)	(Greifinger, 2012; Teleki et al., 2011)
Receipt of hepatitis B vaccine or documented immunity for people with hepatitis C infection or HIV	(Teleki et al., 2011)
Pre- and post- HCV RNA testing of people with a diagnosis of hepatitis C who are receiving anti-viral treatment	(Teleki et al., 2011)
Percentage of HIV positive people with viral load counts under 50,000	(Stone et al., 2006)
Percentage of people with HIV/AIDS prescribed potent ARV therapy	(Teleki et al., 2011)
Percentage of people with HIV/AIDS who were prescribed PCP prophylaxis within 3 months of low CD4þ cell count	(Teleki et al., 2011)
Percentage of people with HIV/AIDS for whom a CD4þ cell count or CD4þ cell percentage was performed at least once in the previous 6 months	(Greifinger, 2012; Teleki et al., 2011)
Percentage of new admissions with documented tuberculosis screening in accordance with guidelines	(Teleki et al., 2011)
Percentage of people with positive test for tuberculosis completing curative therapy within 12 months	(Stone et al., 2006)
Percentage of people with syphilis, gonorrhoea or chlamydia receiving medication within 3 days of lab reports	(Greifinger, 2012)
**4. Mental health care**	
***General:***	
Treatment planning for all people with mental health needs	(Greifinger, 2012; Hoge et al., 2009)
Monitoring of medication adherence	(Hoge et al., 2009)
***Condition-specific:***	
*a) Depression*	
Percentage of people meeting criteria for major depressive disorder (MDD)	(Teleki et al., 2011)
Percentage of people with MDD received at least 3 follow up contacts with a mental health practitioner during the acute treatment phase	(Stone et al., 2006; Teleki et al., 2011; Watts, 2015)
Percentage of people who remained on an anti-depressant during the acute treatment phase	(Stone et al., 2006; Teleki et al., 2011; Watts, 2015)
Percentage of people who remained on an anti-depressant during the treatment continuation phase	(Stone et al., 2006; Teleki et al., 2011; Watts, 2015)
*b) Bipolar 1 disorder*	
Percentage of people with bipolar 1 disorder who have evidence of use of a mood stabilizing or antimanic agent during the first 12 weeks of pharmacotherapy treatment	(Teleki et al., 2011; Watts, 2015)
Percentage of people with bipolar 1 disorder with documented lithium levels in the therapeutic range	(Greifinger, 2012; Hoge et al., 2009; Teleki et al., 2011)
Percentage of people on lithium treatment with a record of serum creatinine and TSH	(Greifinger, 2012; Hoge et al., 2009; Teleki et al., 2011)
Percentage of people presenting with depression who were assessed for the prior or current symptoms and/or behaviors associated with mania or hypomania	(Teleki et al., 2011)
Pre- and post-treatment initiation liver function tests for people prescribed valproic acid	(Greifinger, 2012; Hoge et al., 2009)
Percentage of people receiving tegretol whose levels are in the therapeutic range	(Stone et al., 2006)
*c) Schizophrenia*	
Monitoring of abnormal involuntary movements	(Greifinger, 2012; Hoge et al., 2009)
Percentage of people on antipsychotic medication receiving between 300 and 600 CPZ equivalents per day`	(Teleki et al., 2011)
Percentage of people referred to weekly therapy who have received it	(Watts, 2015)
Percentage of people receiving a dosage of antipsychotic medication outside the recommended range whose medical record documents the dosage given	(Teleki et al., 2011; Watts, 2015)
*d) Suicide*	
Annual incidence of suicide	(Teleki et al., 2011)
Percentage of people who attempted suicide who had an MH score of > = 3	(Stone et al., 2006)
Universal screening recommended on admission using validated tool; people deemed at risk should be put on suicide watch and immediately referred to the mental health team. All serious attempts at suicide should be reviewed	(Greifinger, 2012; Hoge et al., 2009)
*e) ADHD*	
Percentage of people treated with stimulant medication who had at least three follow-ups with a prescribing practitioner in the acute treatment phase	(Watts, 2015)
Percentage of people treated with stimulant medication who had at least one follow-up with a prescribing practitioner during the continuation phase	(Watts, 2015)
**5. Physical health conditions care**	
*a) Cardiometabolic*	
Percentage of people with coronary artery disease prescribed antiplatelet or beta-blocker medication	(Teleki et al., 2011)
Percentage of people with chest pain who have an ECG	(Teleki et al., 2011; Watts, 2015)
Monitoring and treatment (e.g. ACE inhibitors, beta-blockers) of people with heart failure	(Teleki et al., 2011)
Percentage of people receiving aspirin or beta-blockers after acute myocardial infarction	(Stone et al., 2006; Teleki et al., 2011)
Cholesterol screening after acute cardiovascular events	(Stone et al., 2006; Teleki et al., 2011)
Percentage of people with atrial fibrillation at high risk of thromboembolism prescribed warfarin	(Teleki et al., 2011)
Number of months in which people on warfarin for atrial fibrillation had at least one International Normalised Ratio measurement	(Greifinger, 2012; Teleki et al., 2011)
Blood pressure monitoring for people with hypertension, diabetes, CKD, coronary arterial disease, cardiovascular disease	(Greifinger, 2012; Raimer & Stobo, 2004; Stone et al., 2006; Teleki et al., 2011; Watts, 2015)
Percentage of people with chronic kidney disease referred for AV fistula	(Teleki et al., 2011)
Offloading (pressure relief) treatment for diabetic foot ulcers	(Teleki et al., 2011; Watts, 2015)
Cholesterol and blood glucose monitoring for people with diabetes / chronic kidney disease	(Bisset & Harrison, 2012; Teleki et al., 2011; Watts, 2015)
*b) Respiratory*	
Percentage of people with persistent asthma referred to outside facility or emergency department	(Stone et al., 2006; Teleki et al., 2011)
Percentage of people with bronchitis not treated with antibiotics	(Watts, 2015)
Percentage of people with asthma evaluated by the primary care provider within the designated follow-up time frames	(Teleki et al., 2011)
Percentage of people with COPD receiving appropriate care	(Stone et al., 2006; Teleki et al., 2011)
Percentage of people with chronic obstructive pulmonary disease (COPD) with spirometry results documented	(Teleki et al., 2011; Watts, 2015)
Number of visits for people with a chronic skin ulcer without a prescription or recommendation to use wet to dry dressings	(Teleki et al., 2011)
*c) Musculoskeletal*	
Back pain assessment of those with diagnosis of back pain	(Teleki et al., 2011; Watts, 2015)
Osteoarthritis assessment	(Teleki et al., 2011)
*d) Screening and prevention*	
Smoking cessation treatment or advice	(Watts, 2015)
Colorectal cancer screening	(Teleki et al., 2011)
Breast cancer screening and follow-up evaluation	(Stone et al., 2006; Teleki et al., 2011; Watts, 2015)
Cervical cancer screening	(Stone et al., 2006; Teleki et al., 2011)
Percentage of people with a history of falls who have a documented care plan for falls	(Teleki et al., 2011)
Retinal screening for people with diabetes	(Stone et al., 2006; Teleki et al., 2011; Watts, 2015)
*e) Reproductive health*	
Pregnancy tests	(Teleki et al., 2011)
Pre-natal care	(Stone et al., 2006; Teleki et al., 2011; Watts, 2015)
Percentage of live births delivered by Caesarean section	(Stone et al., 2006)
Post-natal care	(Stone et al., 2006; Watts, 2015)
*f) Wound care*	
Number of visits for people with a chronic skin ulcer without a prescription or recommendation to use wet to dry dressings	(Teleki et al., 2011)
**6. Substance use**	
Percentage of people with a diagnosed substance abuse disorder receiving Screening, Brief Intervention and Referral to Treatment (SBIRT), group or individual substance abuse treatment	(Watts, 2015)
Average daily rate of inmates receiving substance abuse treatment	(K. N. Wright, 2005)
Opioid use monitoring	(Watts, 2015)
**SECTION 2: ORGANISATION-LEVEL INDICATORS**	
**1. Grievances and adverse events**	
Number of medical grievances filed in a month that are handled at the facility level	(Teleki et al., 2011)
Percentage of prisoner grievances related to health care services found in favour of the prisoner in the past 12 months	(Teleki et al., 2011)
Percentage of grievances appropriately addressed within 5 working days	(Greifinger, 2012)
Percentage of non-emergency grievances resolved within 20 business days	(Watts, 2015)
Percentage of emergency grievances resolved within 10 calendar days	(Watts, 2015)
Number of grievances received and resolved	(Laffan, 2016)
Percentage of adverse events (including deaths) reviewed within 30 days	(Greifinger, 2012; Watts, 2015)
Inappropriate prescribing (i.e. prescribing drugs to be avoided for older people) to people over the age of 65	(Teleki et al., 2011)
**2. Inclusivity**	
Provision of interpreters where needed; sick call slips and patient education in other languages	(Greifinger, 2012)
Assessment for and provision of assistance with the activities of daily living for people with disabilities	(Greifinger, 2012)
**3. Co-ordination and transfers**	
Percentage of people whose medication list was received within 4 h of admission Monday-Saturday 9 am-8 pm, or 24 h outside of that timeframe	(Greifinger, 2012; Watts, 2015)
Percentage of people receiving an off-site service who were seen for a follow-up appointment after an offsite visit	(Greifinger, 2012; Watts, 2015)
Percentage of people discharged from hospital with unique discharge diagnoses	(Watts, 2015)
Percentage of timely routine or urgent medication administration	(Greifinger, 2012; Watts, 2015)
Proportion of people whose health records reviewed within 12 h of transfer	(Watts, 2015)
Completeness of medical-record keeping	(Greifinger, 2012)

### Challenges and constraints of implementing quality assessment in the prison setting

Authors of papers in this review described a range of challenges to the implementation of performance measurement system in the prison setting, including changing demographics of the prison population, the functionality of the data system, staffing and resourcing issues, and challenges to standardising quality of care measurement across different organisations.

#### Changes in prison populations

Prison populations in the US have undergone significant changes in recent years, with an increase of over 700% in the size of their prison population between 1970 and 2009 (Karstedt, Bergin, & Koch, 2019). Although numbers have fallen in the past decade, the US prison population per capita (655 per 100,000) is still the highest in the world (Walmsley, 2018).

In addition to the increase in numbers towards the end of the 20th and the first years of the 21st centuries, the demographics of the prison population have changed. Most notably, the prison population is ageing (Maschi & Viola, 2013; Stürup-Toft et al., 2018) and evidence suggests that the prevalence of chronic conditions in US prisons is increasing (Binswanger, Krueger, & Steiner, 2009; Wilper et al., 2009). Additionally, multi-morbidity may be a problem in the older prison population; 85% of the over 50s in prison are reported to have three or more chronic health conditions, while four out of five people aged 65 years and over have a chronic condition that impacts on their physical function (Kintz, 2013). The changing landscape of prison health needs may require a reevaluation of existing sets of quality indicators to assess the quality of healthcare for co- and multimorbid conditions (Asch et al., 2011).

#### Data system functionality

The inadequacies of existing data systems in the prison setting were highlighted in most of the reviewed sources, with key issues being poor co-ordination and a lack of functionality in key areas such as capture and extraction of data (Castro, 2014; Hoge et al., 2009; Watts, 2015), interface with other prison systems (Damberg et al., 2011), prison pharmacies (Castro, 2014; Teleki et al., 2011) and community health care settings (Watts, 2015). A lack of co-ordination with community health care settings leads to clinicians’ reliance on patient self-report which can compromise measures of prison health care quality. However, integrating prison health systems with those of community healthcare settings can be, as Bisset and Harrison (Bisset & Harrison, 2012) noted, ‘unfamiliar and daunting territory’ (p.3). Inconsistency in data input was also reported as a problem that could adversely affect the reliability of analyses (Bisset & Harrison, 2012; Damberg et al., 2011; Teleki et al., 2011).

The absence of prison-specific benchmark data was also cited as an inhibiting factor to quality assessment (Damberg et al., 2011; Stone et al., 2006; K. N. Wright, 2005). Additionally, the capacity of the data collection system was perceived to be problematic, with requirements to collect data for legal purposes competing with the collection of data for quality monitoring purposes (Teleki et al., 2011; Watts, 2015): Teleki et al. (2011) observed that there are ‘too many metrics being tracked for too many different purposes’ (p.110) which can dilute performance measurement efforts. The same authors also identified difficulties clarifying the numerator and denominator, and a concern that the amount of data for some conditions would be too small to conduct a meaningful analysis.

#### Organisational issues

Some authors highlighted the difference in priorities between the medical staff and the prison administrators (Hoge et al., 2009; Laffan, 2016), noting that healthcare budgets may be managed by people lacking experience of healthcare delivery (Watts, 2015) and that effective quality assessment of healthcare required collaboration between the two systems.

High levels of staff turnover (Hoge et al., 2009) and the need to employ a data analyst to write and run queries (Damberg et al., 2011) were seen as difficulties that could jeopardise attempts to measure the quality of healthcare. In addition, the lack of a feedback loop for staff to gain insights into under-performing services can impede quality improvement activities (Teleki et al., 2011).

A further issue raised is whether standardisation should occur when institutions have varying mission statements, legal structures and populations (K. N. Wright, 2005). Standardisation can also be compromised by the lack of universal agreement on disease management for chronic health conditions.

## Discussion

To the authors’ knowledge, this is the first scoping review on quality indicators and performance measurement for healthcare in the prison setting. While all the evidence sources identified originated from the US, a number of significant issues have been identified with relevance to performance management in prison healthcare systems beyond America. This review found that selection processes varied both in rigour and in stakeholder involvement, with none including patient representation. Secondly, indicators were predominantly process-oriented with few measures of outcomes or structure. Finally, a range of challenges to performance measurement for prison healthcare was identified including the comparability of prison and community populations, limited data functionality and resource constraints.

### Rigour in development

Kötter et al. (2012) have provided a useful systematic review describing and comparing methods of quality indicator development for healthcare delivery. While they affirm that there is no ‘gold standard’ for developing indicators from clinical guidelines, they identify six steps in the rigorous development and implementation of quality indicators: topic selection, guideline selection, extraction of recommendations, quality indicator selection, practice test and implementation. To ensure the establishment of quality indicators that meet certain criteria – relevance to the population, evidence-based, feasible, reliable, understandable, achievable, measureable and amenable to change – selection methods, they argue, should have a high degree of transparency and rigour.

The selection processes identified in this review were largely opaque, with Asch et al.’s (2011) RAND/University of California, Los Angeles (UCLA) modified Delphi approach the most systematic and transparent. Consultation methods in Wright’s (2005), Stone et al.’s (2006), Hoge et al.’s (2009) and Watts (2015) work, while present, were less clearly articulated, with little detail given on the participants or the process. There was no evidence of consultation processes in other published lists of performance measures (Greifinger, 2012; Laffan, 2016; Raimer & Stobo, 2004). Common to all attempts to develop quality indicators or performance measures described in this review, there was no indication that patients had been involved, despite recognition from the RAND research team that patient experience is an important facet of quality assessment efforts (Asch et al., 2011; Damberg et al., 2011; Teleki et al., 2011) and that patient acceptability of a treatment or intervention is a well-established component of quality in both health and behavioural sciences (Gainforth, Sheals, Atkins, Jackson, & Michie, 2016; Maxwell, 1992). Currently, however, there are relatively few examples of patient engagement in prison health care organisation, and greater efforts to meaningfully engage people who’ve lived in prison are warranted.

### Transferability and adaptation

In their conceptualisations of quality in health services, both Kessner et al. (1973) and Maxwell (Maxwell, 1992) highlighted the importance of quality indicators being relevant and appropriate to the population served by the health system. In this group of papers, Stone et al. (Stone et al., 2006) most clearly attempted to gain evidence of the comparability of prison population demographics to those of the community in order to ascertain whether quality indicators used in the community could be utilised in the prison setting, although other authors quoted prevalence statistics of particular health conditions or evidence of poor quality care to substantiate their attempts to create performance measures. While it must be acknowledged that many of the papers were written when the ageing prison population was perhaps less evident, little reference was made to the benefits of including indicators that account for high levels of co- and multi-morbid mental and physical health conditions (Stürup-Toft et al., 2018; Tyler et al., 2019). Additionally, colorectal and cervical cancer screening indicators were only included by a few authors, and none of the papers included in this review incorporated dementia indicators. Little is known about the prevalence of dementia in prison populations (Brooke, Diaz-Gil, & Jackson, 2018), but it is likely that, with increasing numbers of people in prison over the age of 50, and developing awareness that dementia can affect people under 65 years old (Carter, Oyebode, & Koopmans, 2018), prison health services will be required to provide screening and support for people with dementia.

Use of community indicators in prison healthcare services presents opportunities to assess equivalence. The quality of primary care in community general practice in England is monitored by the Quality and Outcomes Framework (BMA & NHS England, 2021); however, reporting on this indicator set is not mandated in English prisons and is hence inconsistent across the sector (N. Wright, Hankins, & Hearty, 2021). In the USA, community healthcare performance measures include the Healthcare Effectiveness Data and Information Set (HEDIS®) and the Uniform Data System (Health Resources and Services Administration, 2021). This latter set may provide particularly useful data since it is reported on by Federally Qualified Health Centers which serve vulnerable communities demographically similar to incarcerated populations. Use of these indicator sets makes it possible to understand *how* healthcare can be compared across populations, but ongoing debates about the interpretation of the equivalence principle mean questions remain about *what* should be compared.

### Equivalence of care or outcomes?

Assessing performance of health services requires a multi-faceted conceptualisation of quality. According to Maxwell (1992), population relevance, effectiveness, efficiency, acceptability, access and equity are all criteria that should be satisfied by quality measurement processes. Access and equity, he notes, are sometimes conflated on the basis of the assumption that inequities are created by unequal access. Maxwell counters this conceptual stance, proposing that inequities caused by, for example, institutionalised racism, cannot be subsumed within the notion of access. In essence, this standpoint about the distinction between access and equity is at the heart of discourse around the equivalence principle, which, it has been argued, is typically interpreted as equivalence of care rather than equivalence of outcome (Birmingham et al., 2006; Charles & Draper, 2012; Exworthy et al., 2011; Jotterand & Wangmo, 2014; Niveau, 2007). The tacit assumption within the notion of the equivalence of care is that the prison population is comparable to the population as a whole - rather than ‘inherently dissimilar’, as Exworthy et al. (2011) (p. 201) would have it - and therefore that the same standard of health services will produce equivalent health outcomes. The greater disease burden experienced by prison populations on account of socioeconomic determinants (Stürup-Toft et al., 2018; Tyler et al., 2019), combined with accelerated physiological aging (Williams et al., 2012), constraints on their autonomy (Jotterand & Wangmo, 2014) and life in an environment not conducive to healthy lifestyle choices (Ginn, 2013), impact the comparability of the prison population to the population as a whole. Hence, to maximise health equity, that is, to improve the health status of people in prison to a level comparable with the non-incarcerated population, the equivalence principle could be expanded to incorporate equivalence of outcomes, which may require health services in prison to exceed, rather than match, those in the community setting (Lines, 2006). Equivalence of outcomes for socially excluded prison populations, however, remains a significant challenge due to the significant socioeconomic barriers to health incarcerated people face.

It is notable that, in this review, the majority of the measures identified were process, rather than outcome measures. This may be due in part to landmark legal proceedings in America in the 20th century (in particular, the case *Estelle v Gamble* in 1976) which identified poor access to care in prison to be a violation of the 8th Amendment, and subsequently triggered a focus on prison healthcare processes (Damberg et al., 2011; Hoge et al., 2009; Raimer & Stobo, 2004; Teleki et al., 2011; Wilper et al., 2009). However, a primary focus on process rather than outcome indicators has been similarly identified in studies of primary care quality indicators in UK community settings (Kronenberg et al., 2017; Ryan & Doran, 2012). Accountability for process is more readily ascribed than for health outcomes, which are subject to a range of confounding factors including medication adherence, lifestyle choices, and unpredictable trajectories of conditions. However, it is reasonable to suggest that people on the receiving end of care are likely to be more interested in outcome – the chance of an improvement in health status, or the risk of further morbidity or mortality - than the proportion of people who received a particular intervention (Asch et al., 2011), and that inclusion of patients in stakeholder consultations may shift the process-outcome indicator balance.

### Structural indicators

Virtually absent from the reviewed papers is the third category of quality indicators described by Donabedian (Donabedian, 1988): structure. Structural indicators relate to the health care setting, and include measures relating to resources such as budgets, clinical spaces, equipment, staff licencing, training and peer review processes. Structure, process and outcome, according to Donabedian (Donabedian, 1988), are causally linked in that quality in terms of structure creates conditions that are conducive to quality processes which are also likely to promote good outcomes, and that a comprehensive picture of quality relies on a combination of all three types of indicators. In this review, structural indicators were rarely included by authors; none of the indicators in Asch et al.’s (2011) or Stone et al.’s (2006) lists related to structure. Only Laffan (2016) and Greifinger (2012) included structural indicators in their lists of performance measures. Structural indicators may receive less focus because human and material resources within the prison setting – for example the number of clinic rooms - may be less within the bounds of influence of the healthcare team, who could not be held accountable. Secondly, while process and outcome indicators provide data at a patient population level, for example, people living with diabetes, structural indicator data is contextual, relating to the setting of health care delivery, and may be of less interest to health care providers trained to prioritise patient need. However, in line with the above argument on the equivalence principle, where increasing health care services could potentially reduce health disparities between the prison and community populations (Lines, 2006), structural indicators, which provide a way to measure, benchmark and monitor the available healthcare resources in the prison environment, may become more apposite.

#### Limitations

This review aimed to identify international research on quality indicators and performance measurement in the prison setting; however, only literature from the US context was identified, even with the use of supplementary searches. We did not identify any reports on indicator development from within correctional or prison healthcare services using academic search strategies, and would encourage transparent reporting of such processes within peer-reviewed literature. The quality of clinical evidence underpinning the listed indicators was not appraised. Articles not published in the English language may have held valuable content that we were not able to access. Although we approached the literature with a critical stance, we did not use formal critical appraisal tools to eliminate any sources from the review, which resulted in considerable variability in quality.

## Conclusion

Developing a robust set of evidence-based indicators will enable prison establishments to monitor quality of care through both internal and external comparisons and to identify areas for improvement. Challenges exist, however. Selecting indicators is complicated by the number of available guidelines, the unique constraints of the prison setting, the functionality and compatibility of the data infrastructure, and community-prison population comparability. Future research should select indicators that can be implemented using routinely-collected data in prison estates. Where possible, indicators that enable comparison with community settings should be included to reveal imbalances between the quality of prison and community healthcare. Prison health care services could consider adopting community indicators that are in operation in their country, such as the Uniform Data Set in the US and the Quality and Outcomes Framework in England. Achieving an appropriate balance of structure, process and outcome indicators would address the dual objectives set out in the Nelson Mandela Rules, and would make progress towards improving both care quality and health outcomes. Finally, selecting measures of performance requires a rigorous, multi-stakeholder approach in which recipients of prison healthcare are represented alongside healthcare commissioners and providers.

## Supplementary Information

**Additional file 1.** Appendix: MEDLINE (Ovid) Search Strategy.

## Data Availability

Data sharing is not applicable to this article as no datasets were generated or analysed during the current study.

## References

[CR1] Arksey H, O'Malley L (2005). Scoping reviews: Towards a methodological framework. International Journal of Social Research Methodology.

[CR2] Asch SM, Damberg CL, Hiatt L, Teleki SS, Shaw R, Hill TE, Benjamin-Johnson R, Eisenman DP, Kulkarni SP, Wang E, Williams B, Yesus A, Grudzen CR (2011). Selecting performance indicators for prison health care. Journal of Correctional Health Care.

[CR3] Begun AL, Early TJ, Hodge A (2016). Mental health and substance abuse service engagement by men and women during community reentry following incarceration. Administration and Policy in Mental Health and Mental Health Services Research.

[CR4] Binswanger IA, Krueger PM, Steiner JF (2009). Prevalence of chronic medical conditions among jail and prison inmates in the USA compared with the general population. Journal of Epidemiology and Community Health.

[CR5] Birmingham L, Wilson S, Adshead G (2006). Prison medicine: Ethics and equivalence. British Journal of Psychiatry.

[CR6] Bisset MM, Harrison EA (2012). Health outcomes in corrections: Health information technology and the correctional health outcome and resource data set.

[CR7] BMA, & NHS England. (2021). *Quality and outcomes framework guidance 2021/22*. Retrieved from London: https://www.england.nhs.uk/wp-content/uploads/2021/03/B0456-update-on-quality-outcomes-framework-changes-for-21-22-.pdf. Accessed 14 Feb 2022.

[CR8] Brinkley-Rubinstein, L. (2013). Incarceration as a catalyst for worsening health. *BMC Health and Justice*, *1*(3). 10.1186/2194-7899-1-3.

[CR9] Brooke J, Diaz-Gil A, Jackson D (2018). The impact of dementia in the prison setting: A systematic review. Dementia..

[CR10] Carter JE, Oyebode JR, Koopmans RTCM (2018). Young-onset dementia and the need for specialist care: A national and international perspective. Aging & Mental Health.

[CR11] Castro, M. E. (2014). *Diabetes screening in inmates: A quality improvement pilot project* (pp. 468). Doctoral Dissertations. https://opencommons.uconn.edu/dissertations/468.

[CR12] Charles A, Draper H (2012). `Equivalence of care’ in prison medicine: Is equivalence of process the right measure of equity?. Journal of Medical Ethics.

[CR13] Coyle A (2004). Prison reform efforts around the world: The role of prison administrators. Pace Law Review.

[CR14] Damberg CL, Shaw R, Teleki SS, Hiatt L, Asch SM (2011). A review of quality measures used by state and federal prisons. Journal of Correctional Health Care.

[CR15] De Viggiani N (2007). Unhealthy prisons: Exploring structural determinants of prison health. Sociology of Health & Illness.

[CR16] Donabedian A (1988). The quality of care: How can it be assessed. Journal of the American Medical Association.

[CR17] Exworthy T, Wilson S, Forrester A (2011). Beyond equivalence: prisoners' right to health. The Psychiatrist.

[CR18] Fazel S, Hayes AJ, Bartellas K, Clerici M, Trestman R (2016). Mental health of prisoners: Prevalence, adverse outcomes, and interventions. The Lancet Psychiatry.

[CR19] Gainforth HL, Sheals K, Atkins L, Jackson R, Michie S (2016). Developing interventions to change recycling behaviors: A case study of applying behavioral science. Applied Environmental Education & Communication.

[CR20] Ginn S (2013). Promoting health in prison. British Medical Journal.

[CR21] Greenhalgh, T., Howick, J., & Maskrey, N. (2014). Evidence-based medicine: A movement in crisis? British medical journal, 348. 10.1136/bmj.g3725 10.1136/bmj.g3725 PMC4056639 24927763

[CR22] Greifinger RB (2012). Independent review of clinical health services for prisoners. International Journal of Prison Health.

[CR23] Halachmi A (2002). Performance measurement, accountability and improved performance. Public Performance and Management.

[CR24] Health Resources and Services Administration. (2021). Uniform Data System Reporting Requirements for 2021 Health Center Data. Retrieved from https://bphc.hrsa.gov/sites/default/files/bphc/datareporting/pdf/2021-uds-manual.pdf. Accessed 14 Feb 2022.

[CR25] Hoge SK, Greifinger RB, Lundquist T, Mellow J (2009). Mental health performance measurement in corrections. International Journal of Offender Therapy & Comparative Criminology.

[CR26] Hopkin G, Evans-Lacko S, Forrester A, Shaw J, Thornicroft G (2018). Interventions at the transition from prison to the Community for Prisoners with mental illness: A systematic review. Administration and Policy in Mental Health and Mental Health Services Research.

[CR27] Ismail N, de Viggiani N (2018). How do policymakers interpret and implement the principle of equivalence with regard to prison health? A qualitative study among key policymakers in England. Journal of Medical Ethics.

[CR28] Jotterand F, Wangmo T (2014). The principle of equivalence reconsidered: Assessing the relevance of the principle of equivalence in prison medicine. The American Journal of Bioethics : AJOB.

[CR29] Karstedt S, Bergin T, Koch M (2019). Critical junctures and conditions of change: Exploring the fall of prison populations in US states. Social & Legal Studies.

[CR30] Kessner DM, Kalk CE, Singer J (1973). Assessing health quality - the case for tracers. The New England Journal of Medicine.

[CR31] Kinner SA, Young JT (2018). Understanding and improving the health of people who experience incarceration: An overview and synthesis. Epidemiologic Reviews.

[CR32] Kintz KE (2013). Quality measures in correctional health care.

[CR33] Kötter T, Blozik E, Scherer M (2012). Methods for the guideline-based development of quality indicators - a systematic review. Implementation Science.

[CR34] Kronenberg C, Doran T, Goddard M, Kendrick T, Gilbody S, Dare CR, Aylott L, Jacobs R (2017). Identifying primary care quality indicators for people with serious mental illness: A systematic review. British Journal of General Practice..

[CR35] Laffan S (2016). Evaluation of your medical department. American Jails.

[CR36] Lines R (2006). From equivalence of standards to equivalence of objectives: The entitlement of prisoners to health care standards higher than those outside prisons. International Journal of Prisoner Health.

[CR37] Loeb JM (2004). The current state of performance measurement in health care. International Journal for Quality in Health Care.

[CR38] Mainz J (2003). Defining and classifying clinical indicators for quality improvement. International Journal for Quality in Health Care.

[CR39] Maschi T, Viola D (2013). The high cost of the international aging prisoner crisis: Well-being as the common denominator for action. The Gerontologist.

[CR40] Maxwell RJ (1992). Dimensions of quality revisited: From thought to action. Quality in Health Care.

[CR41] McCann LJ, Peden J, Phipps E, Plugge E, O'Moore ÉJ (2019). Developing gender-specific evidence-based standards to improve the health and wellbeing of women in prison in England: A literature review and modified eDelphi survey. International Journal of Prisoner Health.

[CR42] McLeod KE, Butler A, Young JT, Southalan L, Borschmann R, Sturup-Toft S, Dirkzwager A, Dolan K, Acheampong LK, Topp SM, Martin RE, Kinner SA (2020). Global prison health care governance and health equity: A critical lack of evidence. American Journal of Public Health.

[CR43] Niveau G (2007). Relevance and limits of the principle of “equivalence of care” in prison medicine. Journal of Medical Ethics.

[CR44] Omolade, S. (2014). *Analytical summary 2014: The needs and characteristics of older prisoners: Results from the surveying prisoner crime reduction (SPCR) survey*. London: Ministry of Justice (UK).

[CR45] Peters MDJ, Godfrey C, McInerney P, Munn Z, Tricco AC, Khalil H, Aromataris E, Munn Z (2020). Scoping reviews. JBI manual for evidence synthesis: JBI.

[CR46] Raimer BG, Stobo JD (2004). Health care delivery in the Texas prison system: The role of academic medicine. Journal of the American Medical Association.

[CR47] Rushforth, B., Stokes, T., Andrews, E., Willis, T. A., McEachan, R., Faulkner, S., & Foy, R. (2015). Developing ‘high impact’ guideline-based quality indicators for UK primary care: A multi-stage consensus process. *BMC family practice, 16*, *16*. 10.1186/s12875-015-0350-6. 10.1186/s12875-015-0350-6 PMC4624600 26507739

[CR48] Ryan AM, Doran T (2012). The effect of improving processes of care on patient outcomes: Evidence from theUnited Kingdom's quality and outcomes framework. Medical Care.

[CR49] Stone TT, Kaiser RM, Mantese A (2006). Health care quality in prisons: A comprehensive matrix for evaluation. Journal of Correctional Health Care.

[CR50] Stürup-Toft KA, O'Moore ÉJ, Plugge EH (2018). Looking behind the bars: Emerging health issues for people in prison. British Medical Bulletin.

[CR51] Teleki SS, Damberg CL, Shaw R, Hiatt L, Williams B, Hill TE, Asch SM (2011). The current state of quality of care measurement in the California Department of Corrections and Rehabilitation. Journal of Correctional Health Care.

[CR52] Thomas E, Wang E, Curry L, Chen P (2016). Patients' experiences managing cardiovascular disease and risk factors in prison. Health & Justice.

[CR53] Tricco AC, Lillie E, Zarin W, O'Brien K, Colquhoun H, Kastner M (2016). A scoping review on the conduct and reporting of scoping reviews. BMC Medical Research Methodology.

[CR54] Tricco AC, Lillie E, Zarin W, O'Brien KK, Colquhoun H, Levac D, Moher D, Peters MDJ, Horsley T, Weeks L, Hempel S, Akl EA, Chang C, McGowan J, Stewart L, Hartling L, Aldcroft A, Wilson MG, Garritty C, Lewin S, Godfrey CM, Macdonald MT, Langlois EV, Soares-Weiser K, Moriarty J, Clifford T, Tunçalp Ö, Straus SE (2018). PRISMA extension for scoping reviews (PRISMA-ScR): Checklist and explanation. Annals of Internal Medicine.

[CR55] Tyler N, Miles HL, Karadag B, Rogers G (2019). An updated picture of the mental health needs of male and female prisoners in the UK: Prevalence, comorbidity, and gender differences. Social Psychiatry and Psychiatric Epidemiology.

[CR56] United Nations General Assembly (2015). *United Nations Standard Minimum Rules for the Treatment of Prisoners (the Nelson Mandela Rules) : resolution / adopted by the General Assembly, 8 January 2016, A/RES/70/175*. Available at: https://www.refworld.org/docid/5698a3a44.html. Accessed 30 June 2021.

[CR57] Walmsley, R. (2018). *World Prison Population List*. Retrieved from https://www.prisonstudies.org/sites/default/files/resources/downloads/wppl_12.pdf. Accessed 14 Feb 2022.

[CR58] Watts B (2015). Development of a performance-based RFP for correctional health Care Services in Vermont.

[CR59] Williams BA, Stern MF, Mellow J, Safer M, Greifinger RB (2012). Aging in correctional custody: Setting a policy agenda for older prisoner health care. American Journal of Public Health.

[CR60] Willis TA, West R, Rushforth B, Stokes T, Glidewell L, Carder P (2017). Variations in achievement of evidence-based, high-impact quality indicators in general practice: An observational study. PLoS One.

[CR61] Wilper AP, Woolhandler S, Wesley Boyd J, Lasser KE, McCormick D, Bor DH, Himmelstein DU (2009). The health and health care of US prisoners: Results of a nationwide survey. American Journal of Public Health.

[CR62] World Health Organisation (2019). Status report on prison health in the WHO European region.

[CR63] Wright KN (2005). Designing a national performance measurement system. The Prison Journal.

[CR64] Wright N, Hankins F, Hearty P (2021). Long-term condition Management for Prisoners: Improving the processes between community and prison. BMC Family Practice.

