# Supplementary material for: Quality indicators and performance measures for prison healthcare: a scoping review
Source: Health Justice. 2022 Mar 7;10:13. doi: 10.1186/s40352-022-00175-9 (PMC8902782; doi:10.1186/s40352-022-00175-9)
Supplement: Supplementary file 1 — Additional file 1. Appendix: MEDLINE (Ovid) Search Strategy. [file 40352_2022_175_MOESM1_ESM.docx]

**Appendix: MEDLINE (Ovid) Search Strategy**

1 exp Primary Health Care/

2 general practitioners/

3 physicians, primary care/

4 general practice/

5 Family Practice/

6 Community Health Services/

7 Community Health Nursing/

8 ((general or family) adj (practice* or practitioner* or physician* or doctor* or nurs* or dentist*)).tw.

9 GP*.tw.

10 (primary adj4 (care or health* or service* or center* or centre* or practice*)).tw.

11 Nurse Clinicians/

12 Nurse Practitioners/

13 nurse*.tw.

14 Pharmacists/

15 pharmacist*.tw.

16 Physical Therapists/

17 physio*.tw.

18 (physical adj4 therapist*).tw.

19 or/1-18 [Primary care]

20 exp Quality Indicators, Health Care/

21 (quality adj4 (indicat* or measure* or criteria* or indicat* or assurance* or improv*)).tw.

22 ((clinical or performance or safety or process or outcome or prescribing or prevent*) adj4 indicator*).tw.

23 benchmarking.tw.

24 (performance adj4 (evaluat* or measur*)).tw.

25 (performance adj4 (evaluat* or measur* or criteria* or indicat*)).tw.

26 (incentive* adj4 (scheme* or assess* or measure* or outcome*)).tw.

27 "Standard of Care"/

28 (standard* adj2 (healthcare or care)).tw.

29 Quality Indicators, Health Care/

30 "Quality of Health Care"/

31 (quality adj2 (healthcare or care)).tw.

32 patient outcome assessment/

33 (patient adj3 outcome adj (measure* or assessment*)).tw.

34 proms.tw.

35 patient satisfaction/

36 patient preference/

37 (patient* adj3 (experience* or satisf* or preference*)).tw.

38 or/20-37 [Quality indicators]

39 Prisons/

40 Prisoners/

41 ((Secure or correctional) adj2 (unit or units or facility or institution* or facilities or centre* or center*)).tw.

42 (Prison* or jail* or offender* or reoffend* or convict* or inmate* or detainee* or cellmate* or incarcerat* or felon).tw.

43 (Penal or penitentiary or gaol or reformator*).tw.

44 or/39-43 [Prison]

45 19 and 38 and 44
